# 25-Year Storage of Human Choroid Plexus in Methyl Salicylate Preserves Its Antigen Immunoreactivity

**DOI:** 10.5146/tjpath.2022.01581

**Published:** 2023-05-15

**Authors:** Dina A. Sufieva, Elena A. Fedorova, Vladislav S. Yakovlev, Olga V. Kırık, Daria L. Tsyba, Igor P. Grigorev, Dmitrii E. Korzhevskii

**Affiliations:** Department of General and Specific Morphology, Institute of Experimental Medicine, St. Petersburg, Russia

**Keywords:** Choroid plexus, Methyl salicylate, Pineal gland, Human, Immunohistochemistry

## Abstract

***Objective:*** Immunohistochemical investigation of archival histological material is a serious problem, since long-term storage of biological tissues, most often in formalin, leads to a loss of antigenic properties. However, the biological material can also be stored in the clearing agent methyl salicylate. The aim of this study was to assess the antigenicity of the human choroid plexus after extra long-term storage in methyl salicylate.

***Material and Method:*** The study was performed on samples of fixed human choroid plexus (occasionally with attached neighboring pineal gland) stored in either methyl salicylate or paraffin blocks for 25 years. Chromogenic and fluorescence immunohistochemistry of vimentin, GFAP, type IV collagen, β-catenin, α-smooth muscle actin, von Willebrand factor, CD68, mast cell tryptase, TMEM119, and synaptophysin was carried out.

***Results:*** The storage of human choroid plexus in methyl salicylate for 25 years does not impair its histomorphology and preserves the properties of all the antigens assessed, which makes their immunohistochemical visualization possible using both light and fluorescence microscopy. Additionally, we found that long-term storage of human choroid plexus in methyl salicylate does not cause an increase in autofluorescence.

***Conclusion:*** Methyl salicylate can be recommended as a medium for long-term storage of biological tissue, as it provides excellent brain tissue preservation and retains its antigenic properties for up to 25 years.

## INTRODUCTION

Pathological or experimental histological specimens cannot be processed and examined immediately after they are obtained in many cases, and they are then are exposed to long-term storage in a fixative, most often formalin. As a rule, prolonged storage in the formalin does not impair the histomorphology of brain specimens, but the quality of some histological staining is worsened (1). Moreover, prolonged fixation in formalin can result in the irreversible loss of immunoreactivity for many antigens (1–6). However, tissue specimens can be preserved long-term not only in a fixative, but also in methyl salicylate. Methyl salicylate, the main component of wintergreen oil, is a commercially available, strong-smelling organic ester that is used in histological and histopathological laboratories as a perfect substitute for xylene at the clearing stage of preparation processing (7–9). As an excellent clearing agent, it is especially recommended for bone and muscle tissues that are adversely affected by conventional clearing with xylene (10).

Previously, it has been shown that prolonged storage (up to three years) of rat brain specimens in methyl salicylate had no detectable effect on the immunoreactivity of common markers of normal and cancer brain cells, such as neuronal nuclear protein, neuron-specific enolase, glial fibrillary acidic protein (GFAP), vimentin, nestin, and doublecortin (11). These data encouraged us to conduct the present study, in which we assess and confirm the suitability of human choroid plexus samples stored in methyl salicylate for twenty-five years to routine histological staining and immunohistochemical revealing of antigens. The results showed excellent histological staining and good immunohistochemical visualization of various brain antigens (vimentin, GFAP, type IV collagen, β-catenin, α-smooth muscle actin, von Willebrand factor, cluster of differentiation 68 (CD68), mast cell tryptase, transmembrane protein 119 (TMEM119), and synaptophysin) by using both light and fluorescence microscopy. Fluorescence-based imaging techniques are often hampered by the background autofluorescence, which can arise from the endogenous sample components such as age pigment lipofuscin, elastin and collagen proteins, serotonin, catecholamines, and some others (12,13). Formaldehyde fixation is also known to induce an intense autofluorescence in samples of paraffin-embedded animal and human tissues making the fluorescence microscopic observation and immunofluorescence analysis more difficult (14,15). Considering the above, we also aimed to investigate whether prolonged storage in methyl salicylate causes an increase in autofluorescence in the choroid plexus samples.

## MATERIALS and METHODS

The study was conducted in accordance with the Declaration of Helsinki of 1975, and had been approved by the local Ethics Committee. Fragments of choroid plexus (some of them containing adjacent pineal gland) from the lateral and fourth ventricles were removed at routine autopsy from 14 persons (12 males and 2 females aged 20-59) in 1995-1996. The specimens were fixed in alcoholic formalin for 1-2 days and dehydrated in ethanol series. Then, some of the samples were embedded in paraffin routinely, and some were immersed into methyl salicylate (Vekton, St. Petersburg, Russia) in 20 ml glass containers and hermetically sealed. The containers were stored in the laboratory at room temperature. In 2021, after washing in three portions of absolute alcohol, the samples were processed in the spin tissue processor (STP 120, Thermo Fisher Scientific, Waltham, MA, USA) and embedded in paraffin by a routine procedure. Then, samples of choroid plexus in paraffin blocks prepared in 1995-96 (immediately after fixation) and 2021 (after storage in methyl salicylate) were cut to obtain 7 μm-thick sections using rotary (RM 2125RT, Leica Microsystems, Wetzlar, Germany) or sliding (Leica SM 2000R, Leica Microsystems, Wetzlar, Germany) microtome, mounted on poly-L-lysine-coated (Polysine™, Menzel-Gläser, Braunschweig, Germany) glass slides. For histological and immunohistochemical staining, the sections were deparaffinized with xylene, rehydrated in descending gradient of ethanol, and rinsed in distilled water.

Hematoxylin and eosin staining was performed according to the commonly established procedure. For immunohistochemistry, the heat-induced antigen retrieval was performed in modified citrate buffer, pH 6.1 (S1700, Agilent-Dako, Santa Clara, CA, USA) for 25 min at 90ºC. Endogenous peroxidase was quenched by incubation in 3% aqueous solution of hydrogen peroxide for 10 minutes. After washing three times in the phosphate-buffered saline, pH 7.4, the sections were pretreated with the blocking solution (Protein Block, Spring Bioscience, Pleasanton, CA, USA) for 10 min at room temperature. Then, the sections were incubated with the primary antibodies against the following antigens: GFAP, vimentin, synaptophysin, CD68, TMEM119, mast cell tryptase, type IV collagen, α-smooth muscle actin, and von Willebrand factor. Information regarding the panel of antibodies tested in this study and their dilution is presented in Table 1. These antigens are specific markers of astrocytes, microglia, macrophages, synapses, mast cells, endothelial cells, basement membrane of vessels, and smooth muscle cells applicable for both normal and cancer tissues.

**Table 1:** Specification of the primary antibodies used in this study.

**Antibody**	**Clone**	**Manufacturer**	**Dilution**
Vimentin	SP20	Spring Bioscience, Pleasanton, CA, USA	1:200
GFAP	Polyclone	Agilent-Dako, Santa Clara, CA, USA	RTU
CD68	PG-M1	Agilent-Dako, Santa Clara, CA, USA	1:100
TMEM119	Polyclone	Abcam, Cambridge, UK	1:1000
Mast cell tryptase	AA1	Agilent-Dako, Santa Clara, CA, USA	RTU
β-Catenin	Polyclone	Abcam, Cambridge, UK	1:100
Synaptophysin	SY38	Abcam, Cambridge, UK	1:30
Type IV collagen	CIV22	Agilent-Dako, Santa Clara, CA, USA	RTU
α-Smooth muscle actin	1A4	Agilent-Dako, Santa Clara, CA, USA	1:100
Von Willebrand factor	Polyclone	Agilent-Dako, Santa Clara, CA, USA	1:200-1:400

**RTU:** Ready to Use.

To visualize the primary antibodies using light microscopy the following reagents were applied: for rabbit antibodies, Reveal Polyvalent HRP DAB (Spring Bioscience, Pleasanton, CA, USA); for mouse antibodies, MACH 2 Mouse HRP-Polymer (BioCare Medical, Pacheco, CA, USA). The peroxidase label was detected using diaminobenzidine chromogen (DAB+; Agilent-Dako, Santa Clara, CA, USA). After immunohistochemical reactions, some sections were counterstained with hematoxylin or alcian blue. For all immunohistochemical reactions, the control reactions recommended by the antibody manufacturer were performed.

For detection of the primary antibodies by confocal laser microscopy the following reagents were applied: for rabbit antibodies, Reveal Polyvalent HRP DAB (Spring Bioscience, Pleasanton, CA, USA) and anti-HRP Fab-fragment of goat immunoglobulin conjugated with Cy3 fluorochrome (1:100, Jackson ImmunoResearch, West Grove, PA, USA) with emission maximum (λem=569 nm) in red light or anti-rabbit Fab-fragment of donkey immunoglobulin conjugated with a Rhodamine Red-X (RRX) fluorochrome (1:50, Jackson ImmunoResearch, West Grove, PA, USA) with emission maximum (λem=590 nm) in red light; for mouse antibodies, biotinylated anti-mouse antibody (VectorLabs, USA) and streptavidin conjugated with a Rhodamine Red-X (RRX) fluorochrome (1:150, Jackson ImmunoResearch, West Grove, PA, USA) with emission maximum (λem=590 nm) in red light. For nuclei counterstaining, SYTOX Green (1:100, Thermo Fisher Scientific, Waltham, MA, USA) was used. The stained sections were coverslipped using fast-drying permanent non-fluorescent mounting medium Cytoseal™ 60 (Richard-Allan Scientific, Kalamazoo, MI, USA).

For examination of sections in transmitted light, the Leica DM750 microscope and ICC50 camera (Leica Microsystems, Wetzlar, Germany) were used. The sections prepared for confocal laser microscopy were examined under a LSM800 confocal laser microscope (Carl Zeiss, Oberkochen, Germany) using Zen-2012 (Carl Zeiss, Oberkochen, Germany) software for image processing.

## RESULTS

### Histology

**Figure 1:**
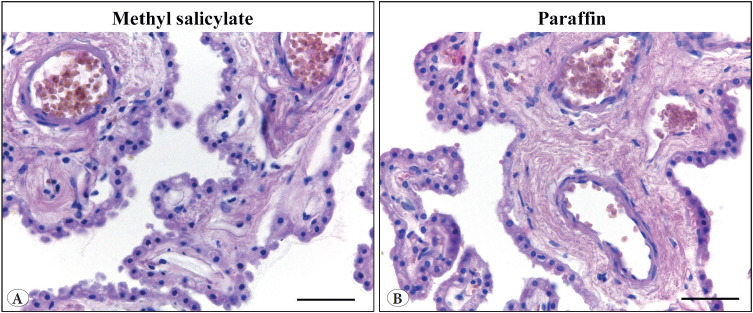
Representative figures of human choroid plexus after 25 years of storage in methyl salicylate **(A),** or paraffin blocks **(B).** Note the well-preserved tissue, crispness of the epithelial cells and blood vessels containing red cells in both methyl salicylate- and paraffin block-stored material. Hematoxylin and eosin staining. x40. Scale bar corresponds to 50 μm.

The primary assessment of the choroid plexus tissue was carried out on preparations stained routinely with hematoxylin and eosin (Figure 1). All tissue samples showed good preservation without visual manifestations of autolysis or tissue compression. The cell nuclei were stained in blue-violet color. The cytoplasm of epithelial cells of the choroid plexus and the smooth muscle cells in the blood vessel walls showed moderate oxyphilia. Red-brown erythrocytes were visible in the lumen of the vessels. Connective tissue fibers were pink in color. No differences in tinctorial properties were found between the tissue samples stored in paraffin blocks or methyl salicylate.

### Immunohistochemistry

**Figure 2:**
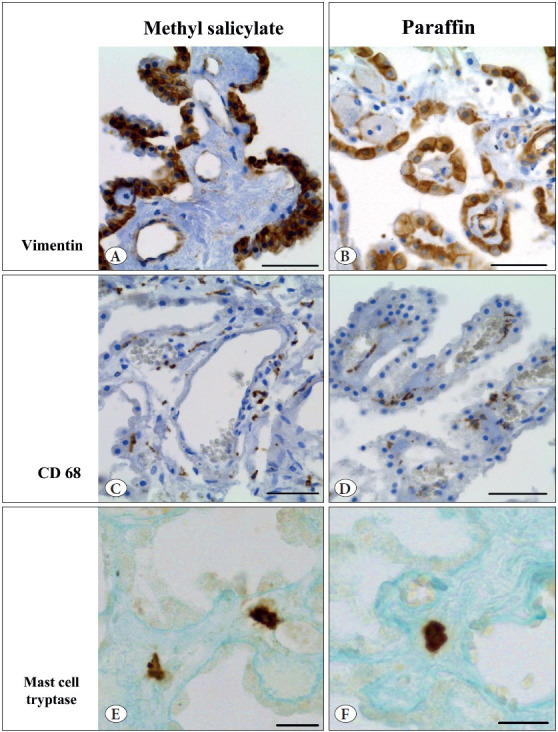
Representative figures of immunostaining for vimentin **(A, B)**, CD68 **(C, D)**, and mast cell tryptase **(E-F)** in the human choroid plexus after 25 years of storage in methyl salicylate (left column), or paraffin blocks (right column). Counterstaining: hematoxylin **(A-D)** or alcian blue **(E-F)**. x40. Scale bar: 50 μm **(A-D)**, 20 μm **(E-F).**

**Figure 3:**
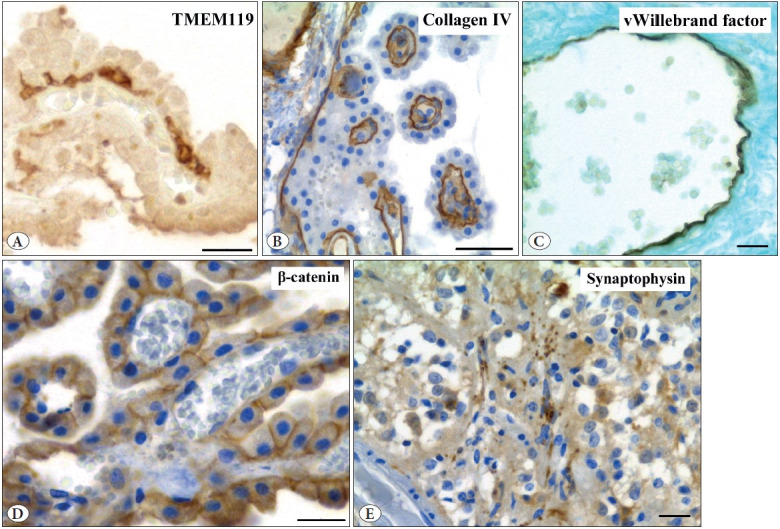
Representative figures of immunostaining for TMEM119 **(A),** type IV collagen **(B)**, von Willebrand factor **(C)**, and β-catenin **(D)** in the human choroid plexus and synaptophysin **(E)** in the human pineal gland after 25 years of storage in methyl salicylate. Note the excellent definition of immunostained structures. Counterstaining: A – none, B, D, E – hematoxylin, C – alcian blue. x40. Scale bar: 20 μm.

**Figure 4:**
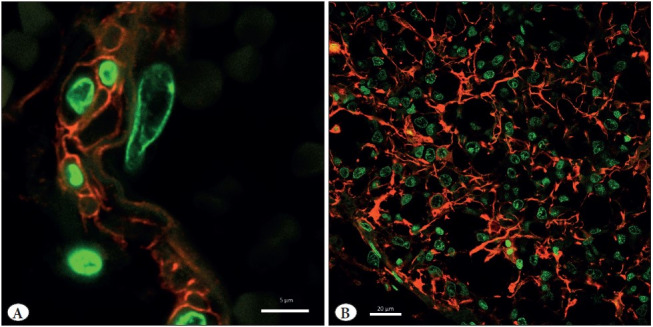
Immunofluorescent imaging of type IV collagen in the human choroid plexus **(A)** and GFAP in the human pineal gland **(B)** after 25 years of storage in methyl salicylate. **A)** type IV collagen (red) and nuclear counterstaining with SYTOX Green (green). **B)** GFAP (red) and nuclear counterstaining with SYTOX Green (green). Confocal laser microscopy. x240 **(A)**, x40 **(B)**. Scale bar: 5 μm **(A)**, 20 μm **(B)**.

All antigens studied preserve their immunoreactivity in the choroid plexus after prolonged storage in methyl salicylate. The used antibodies label their typical targets. The choroid epithelial cells were strongly vimentin-immunopositive and surrounded by β-catenin immunoreactivity (Figure 2, Figure 3). Immunoreaction for CD68, TMEM119, and mast cell tryptase labels macrophages, microglia, and mast cells, respectively (Figure 2, Figure 3). Antibody for α-smooth muscle actin reveals actin inside vascular smooth muscle cells, whereas type IV collagen and von Willebrand factor are concentrated around smooth muscle cells (basal lamina) and in vascular endothelium, respectively (Figure 3). GFAP- and synaptophysin-immunoreactivity was not found in the choroid plexus, but can easily be revealed in the adjacent pineal gland (Figure 3, Figure 4). It is important to note that all immunoreactive structures stain distinctly in the absence of intense background staining. Immunoreaction for all markers was the same regardless of whether it was carried out on the material stored in methyl salicylate or in paraffin blocks. Moreover, the impression was that immunostaining for type IV collagen and vimentin was generally more intense in the choroid plexus after storage in methyl salicylate than in paraffin blocks.

### Confocal Laser Microscopy

Fluorescent immunohistochemistry is also applicable to methyl salicylate-stored material. Representative confocal microscopic image of type IV collagen immunoreactivity is shown in Figure 4. All markers used were visualized by immunofluorescence as clearly and distinctly as by light microscopic immunohistochemistry. No significant autofluorescence was observed. Similarly, a high quality immunofluorescent label was seen in the pineal gland adjacent to the choroid plexus (Figure 4).

## DISCUSSION

The obtained results show for the first time that samples of human choroid plexus and pineal gland preserve their antigenicity after 25-year storage in methyl salicylate. This material is suitable for full-fledged immunohistochemical study of various markers using both light and fluorescence microscopy. The antibodies used in the present study clearly label their typical target structures with minimal background. The localization of vimentin, CD68, tryptase, α-smooth muscle actin, and type IV collagen in the human choroid plexus exposed to prolonged storage in methyl salicylate was similar to that described previously in freshly prepared human choroid plexus (16–20).

To our knowledge, no prior studies have documented the presence of TMEM119 and von Willebrand factor in the choroid plexus of humans or experimental animals (despite an intensive search for TMEM119 in the murine choroid plexus (21)). Thereby, this is the first report on the localization of TMEM119 and von Willebrand factor in choroid plexus. β-Catenin has been described in the choroid plexus of rats, but not in humans (22). Our present observation has shown that its distribution in humans is about the same as in rats.

We failed to detect GFAP- and synaptophysin-immu-nopositive structures in the human choroid plexus. The absence of GFAP expression in the choroid plexus is consistent with the results of other researchers (18). As for synaptophysin, to the best of our knowledge, there are no data on its expression in the choroid plexus. To confirm that the lack of GFAP and synaptophysin is not due to poor antibody quality or improper immunohistochemical technique used, we examined the immunoreactivity of GFAP and synaptophysin in sections of pineal gland tissue presented in some choroid plexus specimens. Well-discernible numerous GFAP- and synaptophysin-immunopositive profiles can be identified in the human pineal gland using the same technique. Therefore, the obtained results indicate the absence of GFAP and synaptophysin expression in the human choroid plexus.

Comparison of material stored in methyl salicylate or in paraffin blocks for twenty-five years revealed an insignificant difference in the intensity and specificity of their immunostaining. Moreover, in some cases, the methyl salicylate-stored choroid plexus appears to show better visualization of the immunohistochemical markers used than paraffin-stored material.

The results obtained demonstrate that not only chromogenic immunohistochemistry, but also fluorescent immunohistochemistry is applicable to choroid plexus samples after long-term storage in methyl salicylate. This material exhibits no signs of increase in background autofluorescence, which is the major drawback to the acquisition of clear images in fluorescent immunohistochemistry. Our observations show that storage of choroid plexus samples in methyl salicylate does not impair the quality of images in a fluorescence microscope in any way.

Currently, formalin is widely used in morphological and pathological laboratories as a conventional fixative and a universal preservative for a long-term storage of histologic material. However, formaldehyde is classified by the International Agency for Research on Cancer as a definitive human carcinogen (Group A1) and poses a significant threat to human health (23,24). This is a significant disadvantage of formalin usage in the laboratory. In comparison to formalin, methyl salicylate is significantly less dangerous for humans: it is harmful if swallowed and may irritate eyes or skin, but this is an inherent property of most other chemicals routinely used in histological and pathological laboratories (25). Moreover, methyl salicylate is used in the clinic as a topical analgesic and anti-inflammatory agent (26). Therefore, the use of methyl salicylate is much safer than the use of formalin.

In addition, prolonged storage of biological samples in formalin can lead to irretrievable loss of many antigens making such material unsuitable for immunohistochemical investigation (1–6). On the contrary, the use of methyl salicylate, according to the data presented here, retains the antigenicity in at least the choroid plexus and the pineal gland after long-term storage. This claim is supported by finding for the first time of TMEM119-, von Willebrand factor- and β-catenin-immunoreactive structures in the human choroid plexus stored in methyl salicylate for 25 years.

Previously, good preservation of the immunoreactivity of a number of antigens has been demonstrated in rat brain samples after prolonged (up to 3 years) storage in methyl salicylate (11). Thereby, methyl salicylate is preferred over formalin if long-term storage of either human or rat brain specimens is required, as it is safe and preserves the immunoreactivity of antigens in brain tissue.

## CONCLUSION

The present study demonstrates for the first time that storage of human choroid plexus and pineal gland in methyl salicylate for 25 years has no detectable influence on histomorphology and quality of standard histological staining. The results obtained show good immunohistochemical visualization of various brain antigens (vimentin, GFAP, type IV collagen, β-catenin, α-smooth muscle actin, von Willebrand factor, CD68, mast cell tryptase, synaptophysin, and TMEM119) by using both light and fluorescence microscopy. Storage in methyl salicylate for 25 years does not intensify the background autofluorescence in human choroid plexus and pineal samples and, thereby, does not impair the quality of immunofluorescence. Therefore, methyl salicylate can be recommended to preserve antigenicity and suitability for histological and immunohistochemical study of the stored material when long-term storage of brain tissue samples is needed.

## Conflict of Interest

The research was conducted in the absence of any financial, personal, academically competitive or intellectual relationships that could be construed as a potential conflict of interest.

## Funding

The study was conducted within the state assignment of the Institute of Experimental Medicine.
